# Supplementary material for: Nanocrystal facet modulation to enhance transferrin binding and cellular delivery
Source: Nat Commun. 2020 Mar 9;11:1262. doi: 10.1038/s41467-020-14972-z (PMC7062909; doi:10.1038/s41467-020-14972-z)
Supplement: Supplementary file 1 — Supplementary Information [file 41467_2020_14972_MOESM1_ESM.pdf]

Supplementary Information for

Nanocrystal Facet Modulation to Enhance Transferrin Binding and Cellular Delivery

By Qi *et al.*

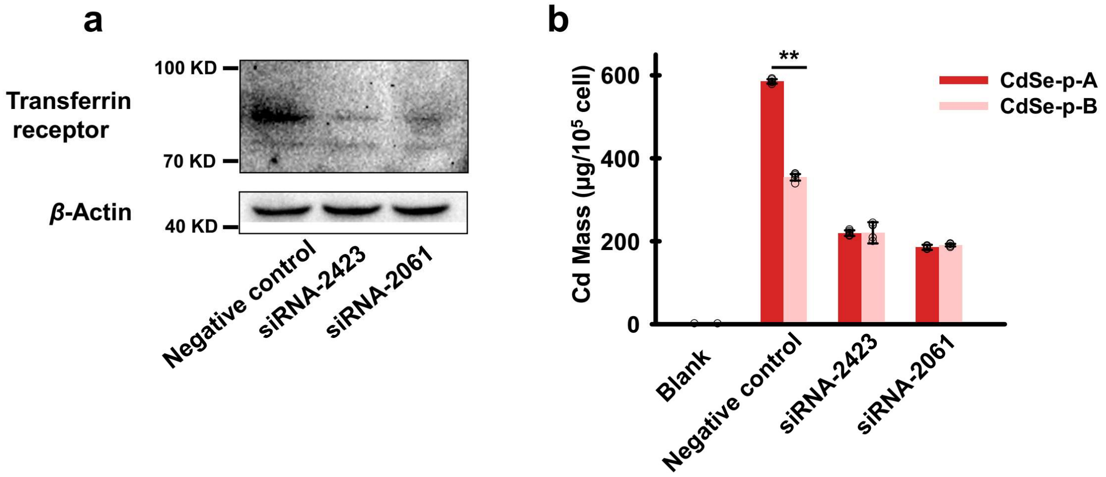

Supplementary Figure 1. (a) Expression of transferrin receptors of HeLa cells transfected by negative control, siRNA-2423 and siRNA-2061, showing that both siRNA-2423 and siRNA-2061 down-regulated the expression of transferrin receptor. Expression of transferrin receptor was independently repeated three times and the results were similar. (b) Cellular content of Cd after the transfected HeLa cells exposed to 40 μg mL^{-1} CdSe nanoparticles for 3 h (CdSe-p-A: dark red bar; CdSe-p-B: light red bar). Data are presented as mean ± SD of five replicate samples by one-way ANOVA (n = 5; $p < 0.0001$ for negative control, and $p = 0.5824$ and 0.1776 for siRNA-2423 and siRNA-2061, respectively). Statistical significance between groups: (**) $p < 0.01$. Source data are provided as a Source Data file.

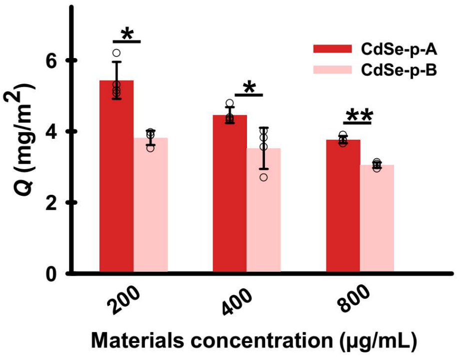

Supplementary Figure 2. The amount of bovine serum albumin adsorbed on different-faceted CdSe nanoparticles. The initial protein concentration was 100 μg mL^{-1} (CdSe-p-A: dark red bar; CdSe-p-B: light red bar). Data are presented as mean ± SD of four replicate samples by one-way ANOVA (n = 4; $p = 0.0208$, 0.0355 and 0.0009 for 200, 400 and 800 μg/mL CdSe nanoparticles, respectively). Statistical significance between groups: (**) $p < 0.01$; (*) $p < 0.05$. Source data are provided as a Source Data file.

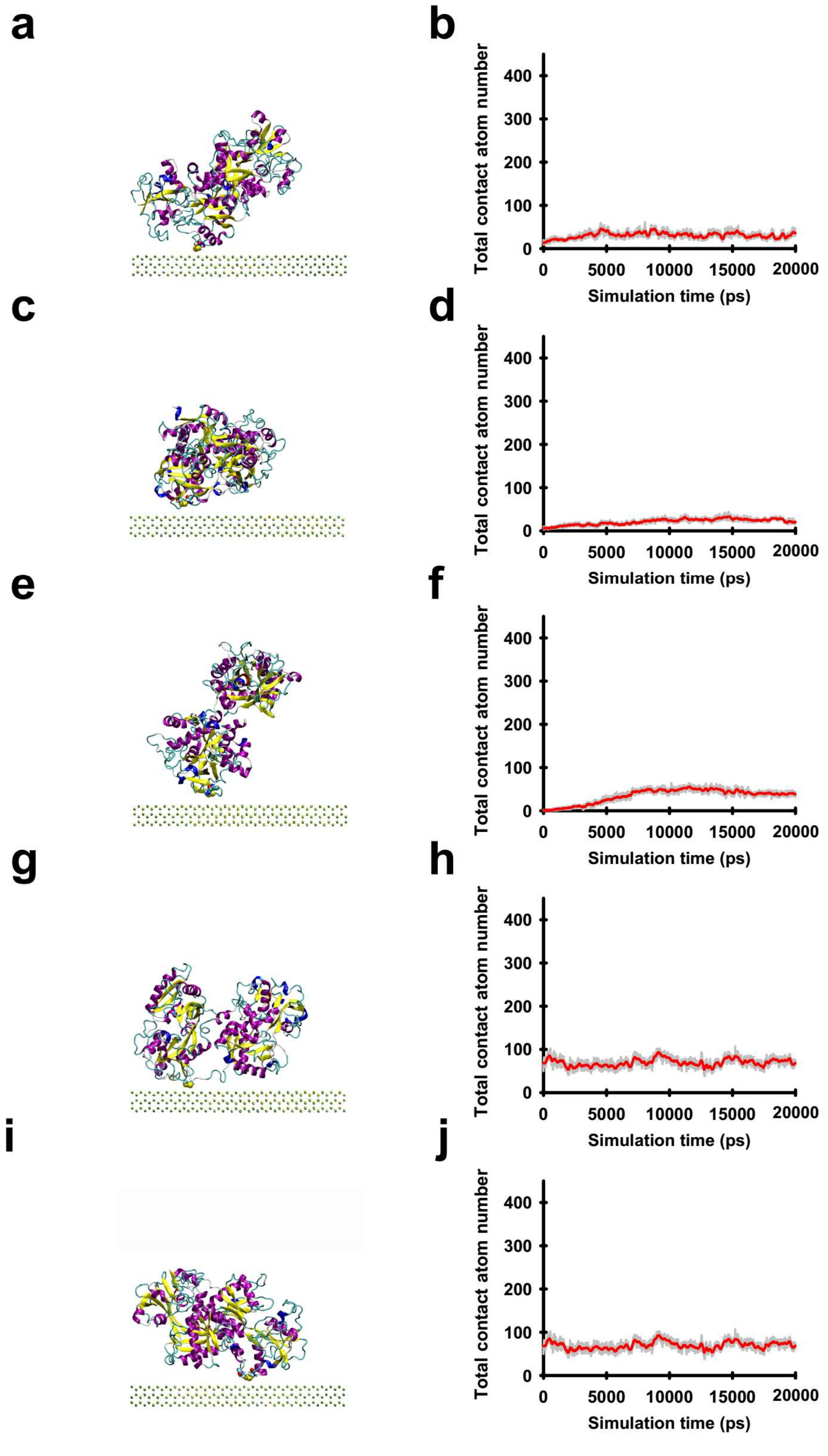

Supplementary Figure 3. Molecular dynamics (MD) simulations of interaction between transferrin and CdSe (100) surface. Different molecule models (a, c, e, g and i) and the corresponding contact atom number (b, d, f, h and j) of transferrin adsorbed on the CdSe (100) facet, calculated by MD simulations. Source data are provided as a Source Data file.

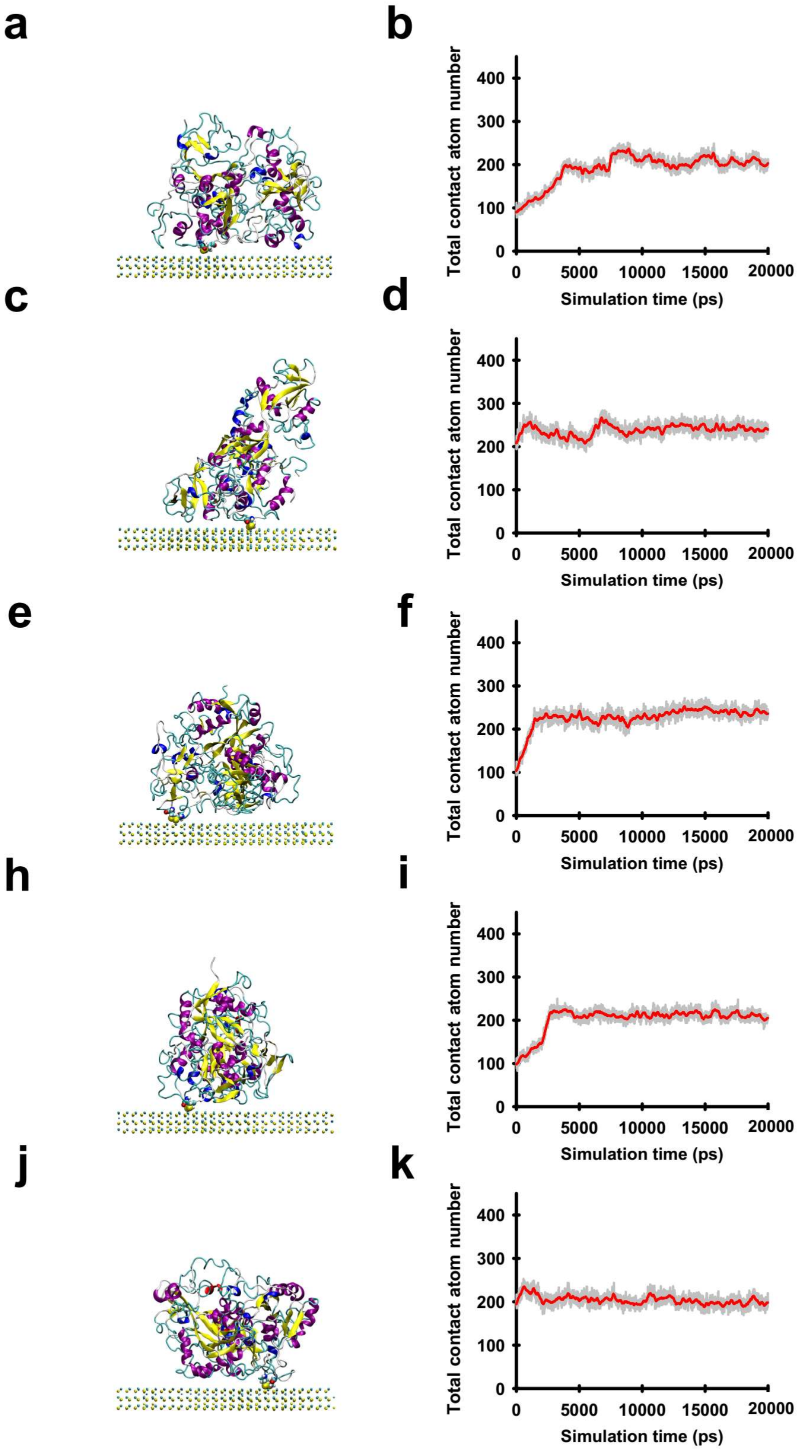

Supplementary Figure 4. Molecular dynamics (MD) simulation of interaction between transferrin and CdSe (002) surface. Different molecule models (a, c, e, g and i) and the corresponding contact atom number (b, d, f, h and j) of transferrin adsorbed on the CdSe (002) facet, calculated by MD simulations. Source data are provided as a Source Data file.

Supplementary Table 1. Selected physicochemical properties of CdSe/CdS nanomaterials

Materials	D_h in DI water (nm)	D_h in PBS with FBS (nm)	ζ *potential* in DI water (mV) [a]	ζ *potential* in PBS with FBS (mV) [a]	Surface area (m^2 g^{-1}) [b]
CdSe-p-A	639.2 ± 10.7 [a]	1782.6 ± 107.6 [a]	-14.8 ± 0.1	-20.4 ± 0.4	22.1
CdSe-p-B	638.4 ± 31.4 [a]	1743.0 ± 215.1 [a]	-17.2 ± 0.4	-20.3 ± 0.8	21.2
CdSe-r-A	707.2 ± 21.8 [a]	1047.0, 1132.0 [c]	-29.5 ± 0.7	-24.5 ± 0.3	8.2
CdSe-r-B	835.4, 863.0 [c]	1121, 1486 [c]	-26.3 ± 3.6	-25.1 ± 0.7	6.0
CdS-r-A	617.6, 540.6 [c]	890.8, 1078.0 [c]	-17.2 ± 0.9	-23.2 ± 0.4	45.7
CdS-r-B	669.2, 659.2 [c]	847.3, 947.9 [c]	-17.6 ± 0.2	-24.7 ± 0.4	30.1

[a] Data are presented as mean ± SD of triplicate samples by one-way ANOVA (n = 3).
[b] Surface area was quantified as Brunauer-Emmett-Teller (BET) specific surface area.
[c] Measurements of duplicate samples are presented (n = 2).

Supplementary Table 2. Parameters and components for SC-ICP-MS analysis

Parameter/Component	Value
Nebulizer	MEINHARD® High Efficiency Nebulizer (HEN)
Spray Chamber	Asperon
Make up Gas (L min^{-1})	0.7
Neb Gas (L min^{-1})	0.54
Plasma Power (W)	1600
Sample Flow Rate (μL min^{-1})	10
Autosampler	Single Cell Micro DX
Agitation	Single Cell Micro DX Autosampler
Sample Loop (μL)	50

Supplementary Table 3. Information for expression of non-thiol transferrin mutant

Gene name	Transferrin（mut）
Plasmid	pET30a
Length of DNA fragments (bp) [a]	2106
Bacterial strain	BL21
Upstream restriction site	EcoR Ⅰ
Downstream restriction site	Xho I
Mass of protein (kD)	84
Tag	HIS

[a] Gene sequence:
GAATTCATGCGTCTGGCTGTTGGGGCTCTGCTGGTTGGTGCAGTTCTGGGTCTGGGTCTGGCAGTACCGGATAAAAC
CGTTCGTTGGGGCGCAGTTTCTGAACACGAAGCAACCAAAGGCCAGAGTTTCCGCGATCACATGAAAAGCGTCATT
CCGTCTGATGGTCCGTCTGTTGCAGGCGTTAAAAAAGCAAGCTATCTGGACGGTATTCGCGCAATTGCAGCAAACGA
AGCAGACGCTGTTACCCTGGATGCAGGTCTGGTTTACGACGCATATCTGGCACCGAATAACCTGAAACCGGTAGTTG
CGGAATTTTACGGCAGCAAAGAAGATCCGCAGACCTTTTATTACGCGGTTGCGGTTGTCAAAAAAGACTCCGGCTTC
CAGATGAACCAGCTGCGCGGTAAAAAATCTGGTCATACCGGTCTGGGTCGTTCTGCTGGCTGGAATATTCCGATTGG
TCTGCTGTACGGCGATCTGCCGGAACCGCGTAAACCGCTGGAAAAAGCAGTTGCGAACTTTTTCTCTGGTTCTGGC
GCACCGGGCGCAGACGGTACCGATTTTCCGCAACTGGGTCAACTGGGTCCGGGTGGCGGCGGTAGTACCCTGAATC
AGTATTTTGGCTACAGCGGCGCATTTAAAGGTCTGAAAGACGGCGCTGGCGACGTTGCGTTCGTCAAACACAGCAC
CATCTTCGAAAACCTGGCGAACAAAGCAGATCGCGATCAGTACGAACTGCTGGGTCTGGATAACACCCGTAAACCG
GTTGACGAGTACAAAGACGGTCATCTGGCACAAGTTCCGAGTCATACCGTTGTTGCTCGTAGTATGGGGGGTAAAGA
GGATCTGATCTGGGAACTGCTGAATCAGGCACAGGAACACTTCGGCAAAGACAAAAGCAAAGAGTTCCAGCTGTTT
AGTAGCCCGCACGGTAAAGATCTGCTGTTCAAAGATTCCGCACACGGTTTTCTGAAAGTTCCGCCGCGTATGGACGC
AAAAATGTACCTGGGCTACGAGTACGTCACCGCAATTCGTAATCTGCGCGAAGGTACCGGTCCGGAAGCACCGACC
GACGAAGGTAAACCGGTTAAATGGGGCGCACTGAGTCATCACGAACGTCTGAAAGGCGACGAGTGGTCTGTTAAC
AGCGTTGGCAAAATTGAAGGCGTTAGCGCAGAAACCACCGAAGACGGTATCGCCAAAATCATGAACGGCGAAGCT
GACGCAATGAGTCTGGACGGCGGCTTTGTTTATATTGCCGGCAAAGGCGGTCTGGTTCCGGTTCTGGCAGAAAATTA
CAACAAAAGCGACAACGGCGAAGACACCCCGGAAGCAGGTTATTTTGCGATTGCGGTTGTCAAAAAATCCGCAAG
CGATCTGACCTGGGACAACCTGAAAGGCAAAAAATCCGGCCATACCGCAGTAGGTCGTACCGCAGGTTGGAATATT
CCGATGGGCCTGCTGTACAACAAAATCAACCACGGCCGCTTCGACGAATTTTTCAGCGAAGGCGGCGCACCGGGTA
GTAAAAAAGATAGTAGTCTGGGCAAACTGGGTATGGGTTCTGGTCTGAATCTGGGCGAACCGAACAACAAAGAAGG
CTATTACGGCTATACCGGCGCATTTCGCGGTCTGGTTGAAAAAGGCGACGTTGCATTCGTCAAACATCAGACCGTCC
CGCAGAATACCGGCGGTAAAAATCCGGATCCGTGGGCAAAAAACCTGAACGAGAAAGACTACGAACTGCTGGGTC
TGGACGGTACCCGTAAACCGGTTGAAGAATACGCAAACGGTCATCTGGCACGCGCACCGAATCACGCAGTTGTTAC
CCGTAAAGACAAAGAAGCGGGCGTTCACAAAATCCTGCGTCAACAGCAGCACCTGTTTGGTAGCAACGTAACCGA
CGGTTCTGGTAACTTTGGTCTGTTCCGCAGCGAAACCAAAGATCTGCTGTTCCGTGACGATACCGTTGGTCTGGCAA
AACTGCACGATCGCAACACCTACGAGAAATACCTGGGCGAAGAATACGTCAAAGCGGTTGGTAACCTGCGTAAAGG
TTCTACCAGTTCTCTGCTGGAAGCAGGTACCTTTCGTCGTCCGCTCGAG
