# Supplementary material for: Nanocrystal facet modulation to enhance transferrin binding and cellular delivery
Source: Nat Commun. 2020 Mar 9;11:1262. doi: 10.1038/s41467-020-14972-z (PMC7062909; doi:10.1038/s41467-020-14972-z)
Supplement: Supplementary file 3 — Description of Additional Supplementary Files [file 41467_2020_14972_MOESM3_ESM.docx]

**Description of Supplementary Files**

**File Name:** Supplementary Data 1

**Description: Ch**aracterization of the hard protein coronas on CdSe-p-A (a), CdSe-p-B (b), CdSe-r-A (c), CdSe-r-B (d), CdS-r-A (e), CdS-r-B (f) nanocrystals, and the proteins in fetal bovine serum (FBS, g). Proteomic analysis was performed using liquid chromatography– mass spectrometry/mass spectrometry (LC−MS/MS). Detailed information were provided according to National Center for Biotechnology Information (NCBInr) database.
