# Supplementary material for: Nanocrystal facet modulation to enhance transferrin binding and cellular delivery
Source: Nat Commun. 2020 Mar 9;11:1262. doi: 10.1038/s41467-020-14972-z (PMC7062909; doi:10.1038/s41467-020-14972-z)
Supplement: Supplementary file 5 — Reporting Summary [file 41467_2020_14972_MOESM5_ESM.pdf]

nature research

Corresponding author(s): Pedro J Alvarez

Last updated by author(s): Jan 29, 2020

Reporting Summary

Nature Research wishes to improve the reproducibility of the work that we publish. This form provides structure for consistency and transparency in reporting. For further information on Nature Research policies, see Authors & Referees and the Editorial Policy Checklist .

Statistics

For all statistical analyses, confirm that the following items are present in the figure legend, table legend, main text, or Methods section.

n/a	Confirmed	
☐	☒	The exact sample size (*n*) for each experimental group/condition, given as a discrete number and unit of measurement
☐	☒	A statement on whether measurements were taken from distinct samples or whether the same sample was measured repeatedly
☐	☒	The statistical test(s) used AND whether they are one- or two-sided<br>*Only common tests should be described solely by name; describe more complex techniques in the Methods section.*
☒	☐	A description of all covariates tested
☒	☐	A description of any assumptions or corrections, such as tests of normality and adjustment for multiple comparisons
☐	☒	A full description of the statistical parameters including central tendency (e.g. means) or other basic estimates (e.g. regression coefficient) AND variation (e.g. standard deviation) or associated estimates of uncertainty (e.g. confidence intervals)
☒	☐	For null hypothesis testing, the test statistic (e.g. *F*, *t*, *r*) with confidence intervals, effect sizes, degrees of freedom and *P* value noted<br>*Give P values as exact values whenever suitable.*
☒	☐	For Bayesian analysis, information on the choice of priors and Markov chain Monte Carlo settings
☒	☐	For hierarchical and complex designs, identification of the appropriate level for tests and full reporting of outcomes
☒	☐	Estimates of effect sizes (e.g. Cohen's *d*, Pearson's *r*), indicating how they were calculated

Our web collection on statistics for biologists contains articles on many of the points above.

Software and code

Policy information about availability of computer code

Data collection	No software was used
Data analysis	Syngistix software (v 4.0), Vienna Ab-initio Simulation Package (VASP, 5.4.4), GROMACS (5.0.4), VMD software (1.9.2), Statistical Product and Service Solutions (SPSS, 20.0)

For manuscripts utilizing custom algorithms or software that are central to the research but not yet described in published literature, software must be made available to editors/reviewers. We strongly encourage code deposition in a community repository (e.g. GitHub). See the Nature Research guidelines for submitting code & software for further information.

Data

Policy information about availability of data

All manuscripts must include a data availability statement. This statement should provide the following information, where applicable:

- Accession codes, unique identifiers, or web links for publicly available datasets
- A list of figures that have associated raw data
- A description of any restrictions on data availability

The data that support the findings of this study are available from the authors on reasonable request. The coordinate of transferrin crystal structure was obtained from RCSB Protein Data Bank (PDB entry: 3V83) (https://www.rcsb.org/structure/3V83). Correspondence and requests for materials should be addressed to TZ and PJA. The source data underlying Figs 1a–c, 2a–c, 3a–d, 4c–f, 4i, 4l, 5c–d, 5g, and 5j and Supplementary Figs 1–4 and Table 1 are provided as a Source Data file.

Field-specific reporting

Please select the one below that is the best fit for your research. If you are not sure, read the appropriate sections before making your selection.

Life sciences study design

All studies must disclose on these points even when the disclosure is negative.

Sample size	Sample size was indicated in each figure legend and determined according to previously established protocols in the literature. e.g., Du et al., Environ. Sci. Tech., 2019 (53); Zhang et al., Nat. Comm., 2019 (10); Zeng et al., Nat. Comm., 2019 (11).
Data exclusions	No data were excluded from the analyses
Replication	All experiments were repeated three to five times and the results appeared to reproducible
Randomization	All samples were randomly allocated into experimental groups
Blinding	The investigators were blinded to group allocation during data collection and analysis

Reporting for specific materials, systems and methods

We require information from authors about some types of materials, experimental systems and methods used in many studies. Here, indicate whether each material, system or method listed is relevant to your study. If you are not sure if a list item applies to your research, read the appropriate section before selecting a response.

Materials & experimental systems

n/a	Involved in the study	
☒	☐	Antibodies
☐	☒	Eukaryotic cell lines
☒	☐	Palaeontology
☒	☐	Animals and other organisms
☒	☐	Human research participants
☒	☐	Clinical data

Methods

n/a	Involved in the study	
☒	☐	ChIP-seq
☒	☐	Flow cytometry
☒	☐	MRI-based neuroimaging

Eukaryotic cell lines

Policy information about cell lines

Cell line source(s)	HeLa cell, purchased from Shanghai Cell Bank of Type Culture Collection of China
Authentication	Provided by Genetic Testing Biotechnology. Nineteen short tandem repeat (STR) loci plus the gender determining locus, Amelogenin, were amplified using the commercially available EX20 Kit from AGCU. The cell line sample was processed using the ABI Prism® 3130 XL Genetic Analyzer. Data were analyzed using GeneMapper® ID v3.2 software (Applied Biosystems). Both positive and negative controls were run and confirmed for each sample submitted.
Mycoplasma contamination	The cell lines were not tested for mycoplasma contamination
Commonly misidentified lines (See ICLAC register)	None
